# The conflicting role highlights the complexity of GSDMs in cancer

**DOI:** 10.3389/fimmu.2025.1531695

**Published:** 2025-03-25

**Authors:** Sijia He, Qian Huang, Jin Cheng

**Affiliations:** ^1^ Cancer Center, Shanghai General Hospital, Shanghai Jiao Tong University School of Medicine, Shanghai, China; ^2^ Department of Oncology, Jiuquan Branch of Shanghai General Hospital, Jiuquan, Gansu, China

**Keywords:** cancer, gasdermins, pyroptosis, tumor microenvironment, anti-cancer therapy

## Abstract

Gasdermins (GSDMs) are an important family of proteins that have received extensive attention in tumor research in recent years. They directly induce tumor cell death by mediating pyroptosis and also regulate the recognition and clearance of tumor cells by the immune system by affecting the microenvironment. Therefore, it is of great significance to investigate the role of GSDMs in tumor development and tumor microenvironment. It can not only reveal new mechanisms of cancer development, but also provide theoretical basis for the development of novel anti-tumor therapeutic strategies. This literature review aims to systematically summarize the dual roles of GSDMs in tumor development and their interactions with the tumor microenvironment, and to focus on the importance of GSDM-mediated pyroptosis in anti-cancer therapy, with a view to providing guidance for future research directions.

## 1 Introduction

Gasdermins (GSDMs) are a family of proteins that have attracted attention due to their ability to form pores in cell membranes, leading to a type of programmed cell death (pyroptosis) characterized by cell swelling and lysis (1, 2). Physiologically, GSDMs play a crucial role in the immune response, and their ability to penetrate cell membranes not only triggers cellular pyroptosis, but also releases pro-inflammatory cytokines, which influence the recruitment and activation of immune cells. This mechanism is essential for fighting against infection and maintaining tissue homeostasis. Pathologically, dysregulated activity of GSDMs is associated with a variety of diseases, including inflammation and cancer (1–3). Recent studies have highlighted their dual functions in cancer, where they can induce tumor cell pyroptosis, enhance anti-tumor immunity, or promote tumor progression by contributing to the inflammatory microenvironment (1, 3). For instance, decreased expression of gasdermin D (GSDMD) was reported to promote gastric cancer cell growth via activating extracellular signal-regulated kinases (ERK), signal transducer and activator of transcription 3 (STAT3) and phosphatidylinositol 3-kinase (PI3K)/protein kinase B (PKB) signaling pathways (4). However, knockdown of GSDMD suppresses non-small-cell lung cancer (NSCLC) cell proliferation by inhibiting EGFR (epidermal growth factor receptor)/AKT signaling (5). Understanding these complex interactions is crucial for the development of novel cancer therapies targeting the GSDMs pathway. The aim of this review is to explore recent findings in the study of GSDMs, highlight their emerging role in the tumor microenvironment (TME), and discuss their potential as targets for innovative therapeutic strategies.

### 1.1 Structure of GSDMs

The GSDM protein family consists of GSDMA, GSDMB, GSDMC, GSDMD, GSDME/DFNA5 (deafness autosomal dominant non-syndromic sensorineural 5), and GSDMF/DFNB59 (deafness autosomal recessive 59)/PJVK (pejvakin). Although 10 members of the GSDM protein family have been identified in mice, only 6 members have been identified in humans, known as GSDM A-F (1). GSDMs share an average of 45% sequence homology, and most are characterized by two highly conserved domains in their structures: the N-terminal domain (GSDM-NT), which is involved in pore formation, and the C-terminal domain (GSDM-CT), which inhibits the N-terminal domain through a common autoinhibitory mechanism (6). However, DFNB59 lacks the C-terminal domain, and its ability to form pores in membranes has not been verified (6).

### 1.2 Expression of GSDMs

GSDMs can be expressed anywhere in the body that is infected and responds to infection, and different GSDMs are selectively expressed at specific mucosal sites in varying abundance. For example, GSDMA is expressed primarily in the skin and gastrointestinal tract, GSDMB is expressed primarily in the lungs, esophagus, gastrointestinal tract, and immune cells, GSDMC is expressed primarily in keratinocytes and gastrointestinal tract, and GSDMD is expressed primarily in the epithelium of the gastrointestinal tract and in the immune system in sentinel cells, macrophages, and dendritic cells (DCs). GSDME can be expressed in the mesenchymal cells-muscle, central nervous system and placental tissue (7).

### 1.3 Regulation of GSDMs

#### 1.3.1 Transcriptional regulation of GSDMs

Under normal conditions, GSDMs are autoinhibited in their inactive state, by folding of the C-terminal domain onto the N-terminal domain, which harbors regions that mediate lipid binding and membrane insertion. Different caspases and proteolytic enzymes can cleave the junction region to release GSDM-NT, which binds to a variety of phospholipids such as phosphatidylinositol phosphates (PtdIns (4)P and PtdIns (4,5)P_2_) and cardiolipin, which are found mainly in cell membranes, mitochondria, organelle membranes, and bacterial cell walls (8). Cryo-electron microscopy studies have shown that the GSDM-NT oligomerizes to form a β-barrel structure that spans the lipid bilayer. This pore is approximately 10-14 nm in diameter, sufficiently large to allow the passage of ions and small molecules, which contributes to the rapid cell lysis observed in pyroptosis. It is therefore clear that understanding how GSDMs are regulated will help us to regulate cellular pyroptosis as well as cytokine release. Transcriptional regulation is a very important regulatory mechanism for proteins, and this is also true for GSDMs (9). It is demonstrated that GSDMA expression is regulated by transforming growth factor-beta (TGF-β) signaling through the transcription factors LIM domain only 1 (LMO1) and Runt-related transcription factor 3 (RUNX3), which bind to the GSDMA promoter (10). GSDMB expression is influenced by elements such as a strong HERV LTR promoter or an Alu element in its promoter region. A regulatory Alu element in the 5’ upstream region of GSDMB has been identified as a positive regulator of its expression. Reporter assays demonstrate that a putative (IKAROS family zinc finger 1) IKZF binding motif within this Alu element is crucial for GSDMB upregulation (11). GSDMC expression is induced by UV irradiation via the (transient receptor potential cation channel subfamily V member 1) TRPV1 channel and the nuclear factor of activated T−cells, cytoplasmic 1 (NFATc1) transcription factor (12). Interferon regulatory factor 2 (IRF2), a key driver of inflammasome responses, plays a critical role in GSDMD transcriptional activation. GSDMD expression is significantly reduced in IRF2-deficient cells and tissues, resulting in decreased IL-1β secretion and pyroptosis inhibition. Mechanistically, IRF2 directly binds to a highly conserved CACT core sequence in the GSDMD promoter region to drive its transcription. Targeting IRF2 or disruption of this binding site blocked both classical and nonclassical inflammatory vesicle signaling (13). The transcription factor specificity protein 1 (Sp1) has been shown to have a critical role in the regulation of GSDME expression. Sp1 promotes the transcription of GSDME through direct binding to approximately 30 base pairs upstream of its transcription start site (-36 to -28). Knockdown or inhibition of Sp1 decreases GSDME expression, thereby reducing chemotherapeutic drug-induced cellular pyroptosis. This process synergizes with STAT3 activity and counteracts DNA methylation, but has little effect on GSDMD-mediated pyroptosis or tumor necrosis factor (TNF)-induced necroptosis (14). Genotoxic stress induces GSDME expression in a p53-dependent manner, as p53 binds to a sequence located in intron 1 (15). In addition, the methylation status of GSDMs promoter affects its expression. Treatment with the DNA methyltransferase inhibitor 5-aza-2’-deoxycytidine (decitabine) led to upregulation of GSDMA expression, which was accompanied by a decrease in promoter methylation, suggesting that promoter methylation plays a key role in regulating GSDMA expression (16). GSDMB overexpression is often caused by gene amplification, while its low expression is due to promoter methylation (17). Hypomethylation of the GSDMC promoter is linked to overexpression in lung adenocarcinoma (LUAD) (18). GSDME is silenced in many cancers due to promoter hypermethylation, which is associated with increased metastasis risk (19, 20).

#### 1.3.2 Post-translational modifications of GSDMs

**Figure 1:**
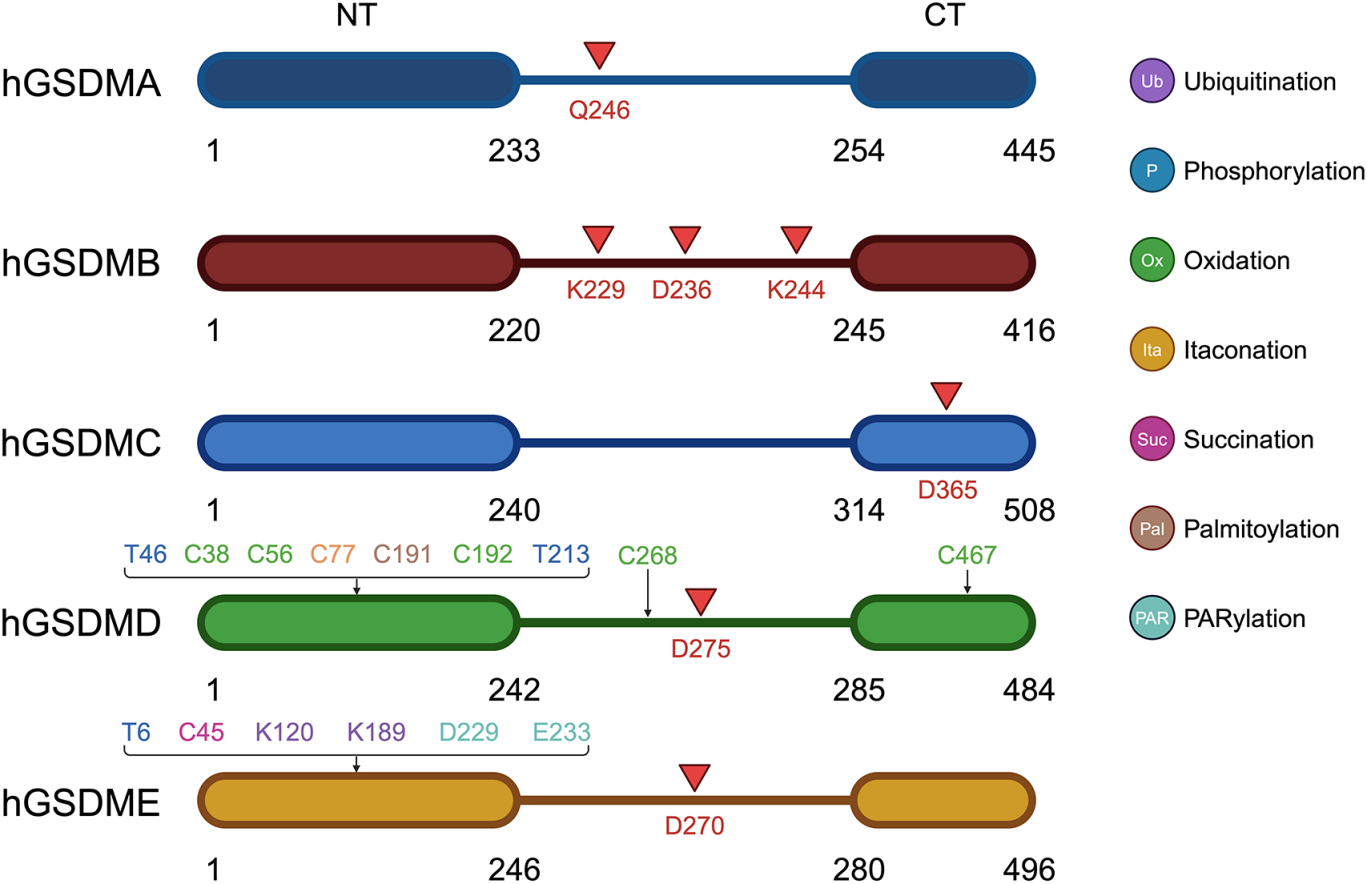
Schematic representation of the structure, cleavage and post-translational modification sites of GSDM A-E. Red arrows point out the cleavage site of GSDMs.

The dynamic regulation of GSDMs activity is further modulated by PTMs and interactions with other cellular proteins (**Figure 1**). Ubiquitination is an important PTM used to direct protein degradation. However, ubiquitination is reversible and can be reversed by a large class of proteases known as deubiquitinating enzymes (DUBs) (21). USP48 is an important member of the USP family of DUBs and involved in modulating the stability of many proteins, such as Mdm2, Gli1, and tumor necrosis factor receptor-associated factor 2 (TRAF2) (22, 23). It was found that deletion of USP48 significantly inhibited tumor cell pyroptosis. USP48 stabilized GSDME to promote pyroptosis by removing K48-linked ubiquitination at K120 and K189 on GSDME (23). Ovarian tumor family deubiquitinase 4 (OTUD4) was found to stabilize GSDME through deubiquitination, enhance tumor cell pyroptosis and increase radiosensitivity. Nasopharyngeal carcinoma (NPC) patients with low OTUD4 and GSDME expression exhibited the worst radiotherapy response and survival (24).

Protein phosphorylation is also a very important PTM that regulates protein function. It was found that AMP-activated protein kinase (AMPK) resists pyroptosis by phosphorylating GSDMD-NT at site S46 and inhibiting its polymerization and pore-forming ability. In an *in vivo* tumor model, AMPK-mediated phosphorylation eliminated the anti-tumor activity of GSDM-NT, leading to accelerated tumor growth (25). AMPK was also able to phosphorylate the T6 site of GSDME, preventing caspase-3-induced GSDME cleavage and inhibiting pyroptosis (26). Phosphorylation of GSDMD-NT T213 residue would inhibit GSDMD-induced membrane pore formation, cytokine release, whereas phosphatase 1 (PP1) was able to pyroptosis through GSDMD dephosphorylation (27).

GSDME was found to be able to induce cell pyroptosis in the absence of cleavage. It was found that DNA damage triggered by intense ultraviolet-C (UVC) irradiation activates PARP1 in the nucleus, forming and releasing poly ADP-ribose (PAR) polymers. These polymers activate PARP5 in the cytoplasm to promote GSDME D229 and E233 PARylation, which triggers conformational changes to deregulate autoinhibition. At the same time, UVC promotes cytochrome c-catalyzed cardiolipin peroxidation, which increases lipid ROS, and PARylated GSDME senses these oxidative stresses, leading to oxidative polymerization and targeting of cellular membranes for perforation, and ultimately, pyroptosis. Simultaneous stimulation of PARylation and oxidation of GSDME can synergistically promote pyroptosis (28).

Cellular redox reactions induce cysteinyl modifications, such as the formation of intramolecular or intermolecular disulfide bonds, which in turn regulate cell signaling activity. Studies have shown that cellular redox status is an important determinant of GSDMD activity. It was found that oxidation of four amino acid residues of GSDMD (C38, C56, C268, and C467) under oxidative stress enhances the cleavage of GSDMD by caspase-1, which in turn promotes pyroptosis, whereas preventing oxidative modification of these residues significantly reduces the efficiency of caspase-1 cleavage of GSDMD (29). Moreover, ROS directly affects GSDMD by oxidatively modifying its C192 site to promote GSDMD polymerization, which results in the formation of membrane pores triggering cellular pyroptosis. In macrophages, GSDMD mutants lacking C192 were unable to efficiently form membrane pores or induce pyroptosis (30).

It was found that dimethyl fumarate (DMF) or endogenous fumarate reacts with key cysteine residues of GSDMD to form S-(2-succinyl)-cysteine, which prevents GSDMD from interacting with caspases, thereby limiting its ability to process, oligomerization, and initiate pyroptosis (31). Additionally, this study found that GSDME processing in *GSDMD*-deficient cells was also blocked by DMF. DMF inhibited GSDMD- and GSDME-driven cell death comparably. DMF and monomethyl fumarate (MMF, a cell-impermeable derivative of fumarate) succinate GSDME at C45. Blocking fumarate hydratase reduced GSDMD-independent (GSDME-dependent) cell death and GSDME- N production (31).

Itaconate is a unique regulatory metabolite induced by toll-like receptor (TLR) stimulation in myeloid cells. Endogenous itaconate is a key regulator of signaling 2 activated by NOD-Like Receptor family Pyrin domain containing 3 (NLRP3) inflammatory vesicles after LPS initiation, thereby establishing tolerance to NLRP3 inflammatory vesicle activation. Accumulation of itaconate in response to prolonged inflammatory stimuli prevented the full activation of caspase-1 and modified GSDMD on C77, suggesting that itaconate is an endogenous pyroptosis regulator (32).

It revealed that the C191 site of GSDMD undergoes S-palmitoylation in order to form membrane pores, and this modification is enhanced by mitochondria-generated ROS. Even the cleavage-deficient form of GSDMD (D275A) can be palmitoylated and induce pyroptosis upon inflammasome stimulation or ROS activation, albeit less efficiently. ZDHHC5 and ZDHHC9 are the major palmitoyltransferases, and inflammasome activation and ROS increase their expression. Other human GSDMs also undergo palmitoylation of their N terminals. This study challenges the conventional view that activation of GSDMD relies solely on cleavage and proposes that reversible palmitoylation is the universal activation switch for pore formation of GSDMD and its family members (33).

## 2 GSDMs in pyroptosis

**Figure 2:**
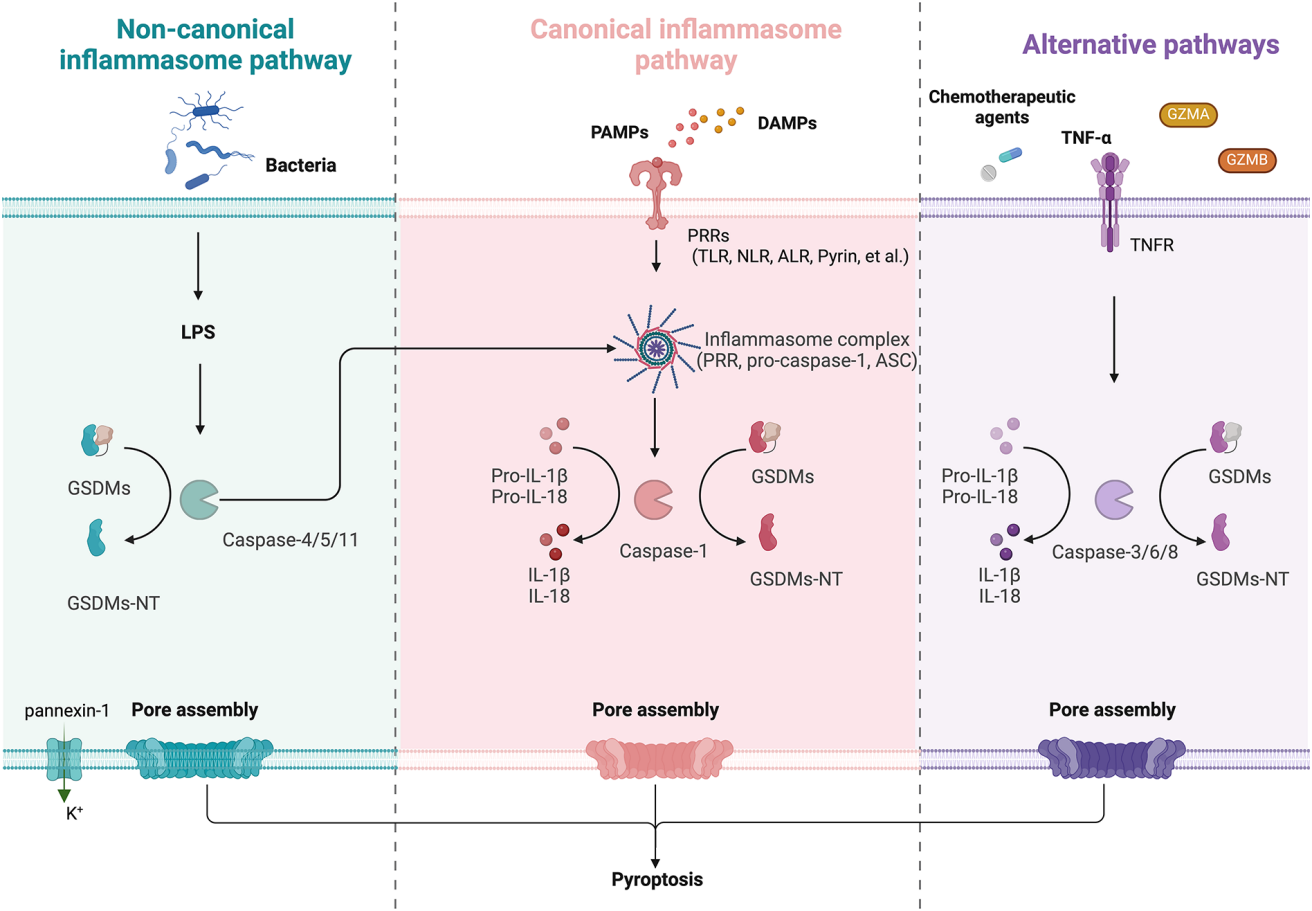
Schematic diagram of GSDM-mediated regulation of pyroptosis, including non-canonical, non-canonical inflammasome pathway and alternative pathways. Left (Non-canonical): LPS directly activates caspase-4/5 in humans or caspase-11 in mice, which cleave GSDMs to form pores. The activated caspase-11 also opens pannexin-1 channels, inducing K^+^ efflux and NLRP3 inflammasome activation, enhancing IL-1β/IL-18 maturation via caspase-1. Middle (Canonical): PRRs detect PAMPs or DAMPs, triggering inflammasome assembly (PRR, ASC, and pro-caspase-1). Active caspase-1 cleaves GSDMs, generating GSDMs-NT and inducing pyroptosis. Right (Alternative): Chemotherapy/TNF-α activate caspase-3/6/8 to cleave GSDMs. GZMA/B directly cleave GSDMs and lead to pyroptosis.

Pyroptosis is a GSDM-mediated programmed cell death (PCD) that was first characterized in 1992 (34). The sites of cleavage by proteases vary among GSDMs (**Figure 1**). The cysteine protease streptococcal pyrogenic exotoxin B (SpeB) triggers pyroptosis in keratin-forming cells by cleaving the Q246 site of GSDMA and releasing the active NT fragment (35). Granzyme A (GZMA) is introduced into cells by electroporation or perforin and cleaves GSDMB at the K244 and K229 site (36, 37), while caspase-1 cleaves GSDMB at the D236 site (38), activating its pore-forming activity and triggering pyroptosis. Caspase-6 and -8 cleaves GSDMC at the D365 site (39). Caspase-3 cleaved GSDMD at D275 and GSDME at D270 (40, 41). Neutrophil elastase (NE) cleaves GSDMD at C268 in humans, which is seven amino acids upstream of the caspase cleavage site D275, and at V251 in mice during bacterial infections, promoting pyroptosis as part of the host defense mechanism. Interestingly, the NE cleavage site is not well conserved between humans and mice, suggesting NE recognizes the tertiary structure of GSDMD rather than a specific sequence. However, its role in the tumor microenvironment (TME) remains less clear and warrants further investigation (42). After activation, the GSDM-NT is released and inserts into the cell membrane to form pores. This pore formation is a critical event in the process of pyroptosis, leading to cell swelling, membrane rupture, and the subsequent release of inflammatory cytokines. To date, two major pyroptosis pathways and several alternative pathways have been elucidated (**Figure 2**).

### 2.1 Canonical inflammasome pathway

The activation of canonical inflammasome pathway relies on pattern-recognition receptors (PRRs, also known as inflammasome sensors) that recognize pathogen-associated molecular patterns (PAMPs) and non-pathogen-associated damage-associated molecular patterns (DAMPs) (43). PRRs are receptors for danger signals that can be activated by many factors, including viruses, fungi, certain drugs, reactive oxygen species (ROS), and endogenous damage signals. Toll like receptor (TLR), intracellular nucleotide-binding oligomeric structural domain (NOD)-like receptor (NLR), absent in melanoma (AIM) 2-like receptor (ALR), and Pyrin are PRRs associated with pyroptosis (40). In general, inflammasome complexes are comprised of a sensor (PRR), an effector (pro-caspase-1), and an adaptor (apoptosis-associated speck-like protein containing a caspase-recruitment domain, also known as ASC or PYCARD). After recognition of DAMPs and/or PAMPs by PRRs, inflammasome assembles and recruits caspase-1, leading to caspase-1 activation, caspase-1 cleavage, and activation of pro-IL-1β and pro-IL-18, which are released extracellularly to trigger an inflammatory response. GSDMD is specifically cleaved by caspase-1 and contributes to the cell membrane perforation (43).

### 2.2 Non-canonical inflammasome pathway

The noncanonical pathway differs from the canonical pathway in that it does not require an inflammasome to activate caspase-1. Lipopolysaccharide (LPS) can directly activate caspase-4/5 in humans or caspase-11 in mice via their C-terminal caspase recruitment domains (CARDs). Active caspase-4/5/11 complexes can cleave GSDMD to generate GSDMD-NT, which perforates the plasma membrane and induces pyroptosis. In addition, LPS-activated caspase-11 opens pannexin-1 (a nonselective large protein channel) and allows K+ efflux, which activates the NLRP3 inflammasome and induces caspase-1 activation and the canonical inflammasome pathway, eventually leading to maturation and release of IL-18 and IL-1β (44). It is worth noting that caspase-4/5/11 cannot cleave pro-IL-1β/pro-IL-18, but they are capable of mediating the maturation and secretion of IL-1β/IL-18 via the NLRP3/caspase-1 pathway (45).

### 2.3 Alternative pathways

Studies have shown that caspase-3/6/8, which is associated with apoptosis, can also induce pyroptosis (46–48). Caspase-3 can induce GSDME-related pyroptosis under certain conditions, such as in the presence of TNF-α, high expression of GSDME or certain chemotherapeutic agents (41). It is found that GSDME is a switch between apoptosis and pyroptosis induced by chemotherapy drugs (47). Pyroptosis occurs when GSDME is highly expressed, and apoptosis occurs when GSDME is expressed at low levels in the presence of chemotherapy drugs. In addition, it has shown that GSDMB can be cleaved by GZMA and GSDME can be cleaved by granzyme B (GZMB) to induce pyroptosis (49).

## 3 Pyroptosis-dependent functions of GSDMs in cancer

Chronic and acute pyroptotic deaths of cancer cells exhibit distinct outcomes. The elusive role of pyroptosis in cancer appears to be context-specific, depending on cell type, genetics and duration of pyroptosis induction.

### 3.1 The anti-tumor effects of GSDMs-induced pyroptosis

By inducing pyroptosis, GSDMs facilitate the release of tumor antigens and pro-inflammatory cytokines, such as IL-1β and IL-18. These released molecules recruit and activate immune cells such as DCs and T-lymphocytes, while further enhancing their ability to recognize and eradicate cancer cells. It is demonstrated that inhibition of CDC20 increases GSDME expression, promoting CD8+ T lymphocytes infiltration, and enhancing immune responses. Apcin, a CDC20 inhibitor, shows synergistic effects with anti-PD1 immunotherapy in murine models of prostate cancer (50). As such, GSDMs contribute to anti-tumor immunity and exhibit tumor suppressive effects. There is growing evidence that chemotherapeutic agents are able to exert antitumor effects by inducing pyroptosis. MEG3 (maternally expressed gene 3)/NLRP3/caspase-1/GSDMD pyroptosis pathway was involved in the cisplatin (DDP)-induced anti-tumor effect of triple-negative breast cancer (TNBC). Knockdown of MEG3 not only blocked the activating effect of DDP on NLRP3/caspase-1/GSDMD pathway-mediated pyroptosis, but also reversed the inhibitory effect of DDP on tumor growth and metastasis (51). It is demonstrated that β5-integrin represses chemotherapy-induced canonical pyroptosis to confer cancer chemoresistance through ASAH2-driven sphingolipid metabolic reprogramming. Chemoresistant cells fail to undergo chemotherapy-induced pyroptosis, which is controlled by β5-integrin. Mechanistically, proteomic and lipidomic analyses indicate that β5-integrin up-regulates sphingolipid metabolic enzyme ceramidase (ASAH2) expression through Src/STAT3 signaling, which then reduces the metabolite ceramide concentration and subsequent ROS production to prohibit chemotherapy-induced canonical pyroptosis (52).

### 3.2 The pro-tumor effects of GSDMs-induced pyroptosis

Conversely, in some cases, GSDMs also promote tumor development. Studies have shown that prolonged activation of GSDMs-mediated pathways may lead to sustained inflammation, thereby supporting tumor proliferation and metastasis. Cancer-associated fibroblasts (CAFs) in breast cancer were found to sense DAMPs and activate the NLRP3 inflammasome pathway, which results in pro-inflammatory signaling and secretion of IL-1β that promotes tumor growth and metastasis, which is attenuated when specifically abrogated by NLRP3 or IL-1β. CAF-derived inflammasomes promote tumor progression and metastasis by modulating the TME towards an immunosuppressive environment and upregulating the expression of adhesion molecules on endothelial cells (53). GSDME-mediated pyroptosis promotes the development of colitis-associated colorectal cancer (CAC) by releasing high mobility group box 1 (HMGB1), which induces tumor cell proliferation and proliferating cell nuclear antigen (PCNA) expression through the ERK1/2 pathway. This finding reveals a previously unrecognized link between pyroptosis and CAC tumorigenesis and offers new insight into CAC pathogenesis (54).

## 4 GSDMs reprogram tumor microenvironment

Recent studies have highlighted the role of GSDMs in the tumor microenvironment (TME), which influences both tumor development and immune responses (1, 3, 55).

### 4.1 Interactions with immune cells

The interplay between GSDMs and various immune cells is the basis for GSDMs to perform their function in TME and mediate immunosurveillance. Upon activation, GSDMs form pores that lead to the release of cytokines. These cytokines are potent chemoattractants that boost the infiltration and activation of DCs, macrophages, and cytotoxic T-lymphocytes (CTLs), thereby favoring anti-tumor immune responses (56). Additionally, IL-1β and IL-18 released during pyroptosis can further polarize the TME towards an immunostimulatory state by promoting DC maturation and T-helper 1 (Th1) differentiation. However, the TME also plays a critical role in shaping the pro-tumorigenic effects of pyroptosis (55). Chronic inflammation resulting from sustained pyroptosis may recruit immunosuppressive cells, such as myeloid-derived suppressor cells (MDSCs), regulatory T cells (Tregs), and tumor-associated macrophages (TAMs) with an M2-like phenotype, which suppress anti-tumor immunity and promote tumor growth. This duality highlights the context-dependent nature of GSDM-mediated pyroptosis, where the balance between pro- and anti-tumor effects is shaped by the immune composition of the TME.

#### 4.1.1 Activation of DCs

The initiation of anti-tumor immunity depends on the stimulation of DCs, which present tumor antigens to naïve T cells and generate effector T cells that can kill cancer cells. Studies have demonstrated that GSDME-derived pyroptosis preferentially activates CD103^+^ and XCR1^+^ type I conventional DCs (cDC1), while activating the number and function of tumor-specific CD8^+^ T cells and decreasing the number of regulatory T cells within the tumor. Depletion of cDC1 or CD4^+^ and CD8^+^ T cells impairs the anti-tumor response and renders experimental mice more susceptible to tumorigenesis (57).

#### 4.1.2 Modulation of macrophage function

Macrophages are responsive to the inflammatory environment created by GSDMs, and GSDMs-mediated pyroptosis, through the release of proinflammatory cytokines, can polarize macrophages toward the M1 phenotype, which is associated with tumor-killing activity and enhanced antigen presentation. This polarization is essential for maintaining an effective immune response against tumor cells. It has been shown that TBD-3C-induced pyroptosis stimulates macrophage M1 polarization, leading to DC maturation and activation of CD8^+^ cytotoxic T lymphocytes (CTLs). The immune response stimulated by pyroptosis could transform the immunosuppressive “cold” TME into an immunogenic “hot” TME, which inhibited the growth of primary pancreatic cancer and distant tumors (58). It has been shown that XBP1 deficiency exacerbates acute liver injury (ALI) by enhancing hepatocyte pyroptosis and macrophage STING signaling, during which stressed hepatocytes activate macrophages by releasing mitochondrial DNA (mtDNA) in a cGAS-dependent manner. XBP1 deficiency impairs mitophagy, increases ROS production and activates the NLRP3/caspase-1/GSDMD pathway. In contrast, restoration of mitophagy by overexpression of PINK1 inhibited macrophage activation and liver injury (59).

#### 4.1.3 Enhancement of T cell responses

GSDMs influence T cell activity indirectly through the modulation of antigen-presenting cells. The pyroptotic release of tumor antigens facilitates their uptake by DCs, leading to enhanced maturation and presentation to T cells. This process boosts T cell priming and activation, which is essential for robust adaptive immune responses. Moreover, GSDM-induced cytokine environments can directly impact T cell proliferation and effector functions. It is reported that interleukin-17A (IL-17A) regulates the tumor microenvironment by promoting pyroptosis and enhancing immune cell recruitment (60). IL-17A not only induces mitochondrial dysfunction, ROS generation and pyroptosis via the ROS/NLRP3/caspase-4/GSDMD pathway, but also increases the infiltration of CD8^+^ T cells in a mouse colon cancer model (60).

#### 4.1.4 Synergy with natural killer cells

Natural killer (NK) cells interact with GSDM-related signaling pathways. The inflammatory environment resulting from GSDM activation enhances NK cell cytotoxicity and promotes cytokine production. This synergistic effect contributes to the elimination of tumor cells and strengthens immune-mediated tumor suppression. Knockdown of GSDME in GSDME-expressing tumors promotes tumor growth, whereas ectopic expression in suppressed tumors inhibits tumor growth. This tumor-suppressive effect of GSDME was associated with enhanced activity of cytotoxic lymphocytes including NK and CD8^+^ T cells. GZMB also induced pyroptosis by cleavage of GSDME. This suggests that GSDME may exert tumor suppressive effects by promoting the process of pyroptosis and enhancing anti-tumor immunity (56). GZMA in NK cells and cytotoxic T lymphocytes was found to cleave GSDMB, activating its pore-forming ability and leading to pyroptosis. Interferon-gamma (IFN-γ) enhances this effect by increasing the expression of GSDMB. Introducing GZMA-cleavable GSDMB into mouse tumor cells promotes tumor clearance (36).

### 4.2 Crosstalk between pyroptosis and other cell death

GSDMs are primarily known for pyroptosis involvement, however, they also serve as a critical nexus between various cell death modalities, offering insights into their broader role in cancer. It was discovered that GSDME-NT not only formed pores in the cell membrane to trigger pyroptosis, but also permeabilized the mitochondrial membrane, released cytochrome c and activated apoptotic vesicles. In cells lacking GSDME, cytochrome c release and caspase-3 activation were significantly reduced, while cell growth was accelerated. In addition, GSDMD-NT similarly permeabilized mitochondria, linking inflammatory vesicle activation to apoptotic vesicle activation. The results suggest that GSDMs can promote apoptotic pathways by affecting mitochondrial function (61). Moreover, caspase-mediated pathways exhibit significant intersections with pyroptosis. For instance, caspase-3, a key executioner of apoptosis, cleaves GSDME and switches apoptosis to pyroptosis and amplifies immune responses through the release of inflammatory cytokines and DAMPs (47). Similarly, caspase-8, traditionally associated with apoptosis initiation, can cleave GSDMC under specific conditions, such as TNF-α stimulation in hypoxic environments, leading to pyroptosis instead of apoptosis (48). These findings demonstrate how caspase-mediated pathways dynamically regulate cell death modes in response to environmental cues. It was found that taxol treatment of NPC cells resulted in pyroptosis with simultaneous activation of caspase-1 and IL-1β, as well as cleavage of GSDMD. Taxol-resistant NPC cells exhibit higher autophagic activity, which attenuates pyroptosis, promotes cell survival, and contributes to resistance. Inhibition of autophagy in these cells restores pyroptosis, sensitizing them to taxol and enhancing its therapeutic efficacy (62). These findings suggest that autophagy acts as a survival mechanism by suppressing pyroptosis, thereby facilitating drug resistance in cancer cells. Targeting autophagy to restore pyroptosis represents a promising strategy to overcome chemoresistance, highlighting the need for further research to elucidate the interplay between autophagy and pyroptosis and its implications in cancer therapy. Necroptosis is another form of programmed necrosis mediated by the receptor-interacting serine/threonine-protein kinase 1 (RIPK1)/receptor-interacting serine/threonine-protein kinase 3 (RIPK3)/mixed lineage kinase domain-like pseudokinase (MLKL) signaling pathway, leading to membrane rupture and inflammation. Like pyroptosis, necroptosis results in the release of DAMPs. Emerging evidence suggests that components of the necroptotic pathway can influence GSDMs activity, potentially through shared signaling molecules or membrane dynamics, although the precise mechanisms remain to be fully elucidated. Ferroptosis is an iron-dependent form of cell death characterized by lipid peroxidation. Although distinct from pyroptosis, recent studies suggest potential interactions between GSDMs and ferroptotic pathways. GSDMs-mediated pore formation may influence iron and ROS dynamics, thereby impacting ferroptotic processes. The interplay between GSDMs and other cell death pathways underscores a complex network of regulatory mechanisms.

### 4.3 Interaction with other TME components

GSDMs also interact with other components of the TME, such as stromal cells and extracellular matrix proteins. These interactions can influence tumor angiogenesis and metastasis. Cytokines such as interleukin-6 (IL-6) and TNF-α, released during pyroptosis, have been implicated in tumor angiogenesis, epithelial-to-mesenchymal transition (EMT), and metastasis (63). For instance, the inflammatory milieu created by GSDM-mediated pyroptosis can either inhibit or promote angiogenesis, depending on the cytokine profile and the cellular context. The researchers used a mouse colon cancer cell line (CT26) overexpressing IL-17F, transplanted it into BALB/c mice, and compared tumorigenic differences between IL-17F knockout and wild-type mice. The results showed that IL-17F inhibited colon tumor growth by suppressing angiogenesis and enhancing immune cell recruitment. In this process, IL-17F promotes endothelial cell pyroptosis by enhancing caspase 4/GSDMD signaling, thereby reducing endothelial cell growth and migration (64).

In summary, GSDMs significantly shape the TME by regulating immune cell dynamics, tumor cell death, and interactions with other microenvironmental components, offering promising avenues for combination therapies. Preclinical models, such as syngeneic mouse models and patient-derived xenografts (PDX), can be employed to study how GSDM activation modulates immune cell infiltration, cytokine release, and tumor immunogenicity. Additionally, organoid cultures and 3D tumor spheroids can be used to recapitulate the TME and investigate the spatial and temporal dynamics of GSDM-mediated effects on stromal cells and immune populations. Clinically, single-cell RNA sequencing (scRNA-seq) and spatial transcriptomics of tumor biopsies before and after treatment with GSDM-activating agents can provide insights into the molecular and cellular changes within the TME. These approaches not only elucidate the mechanistic interplay between GSDMs and the TME but also pave the way for the development of novel combination strategies in cancer therapy.

## 5 Prospects of GSDMs-mediated pyroptosis in anti-cancer therapy

**Figure 3:**
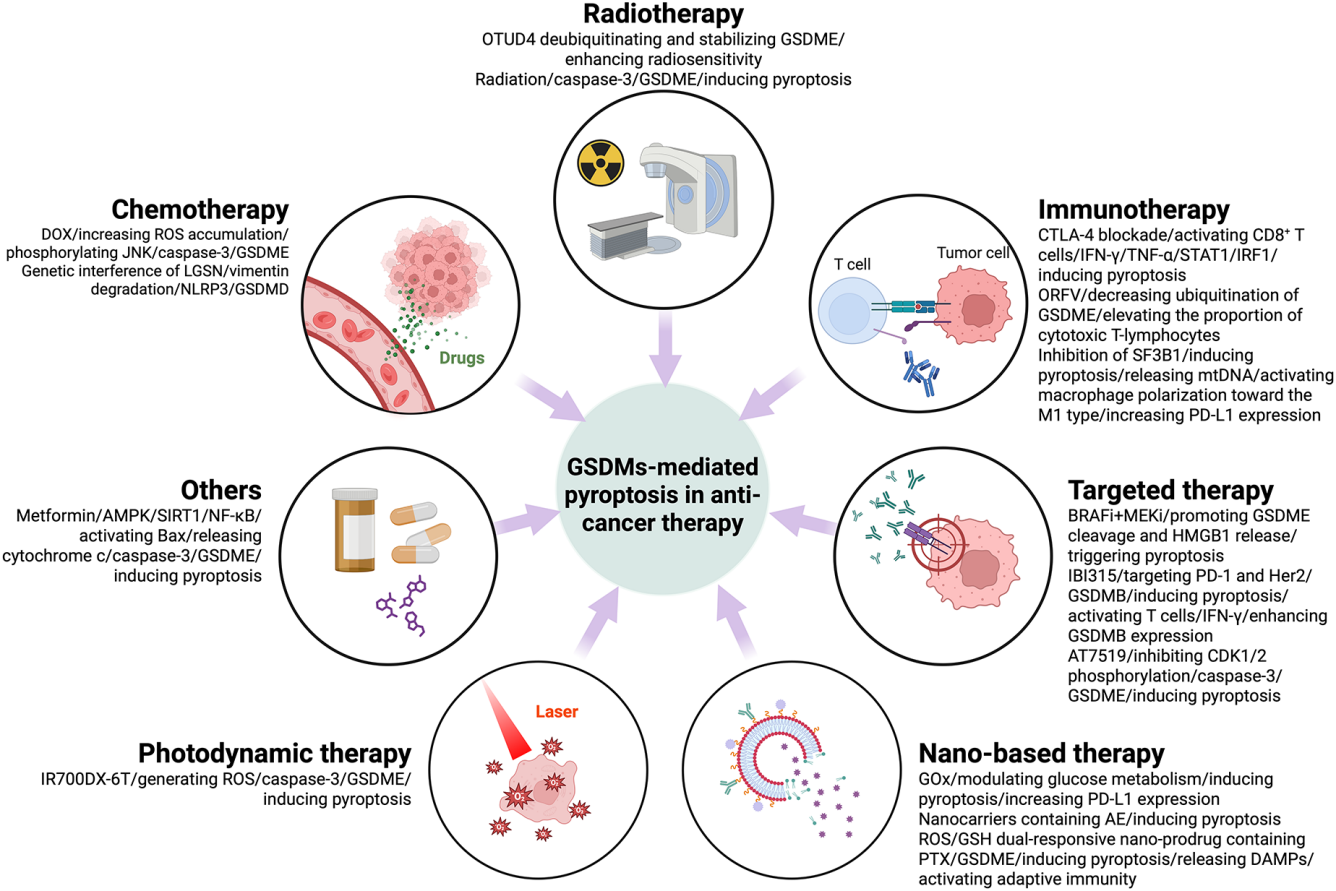
Anti-tumor therapy-induced pyroptosis mediated by GSDMs and signaling pathways that may be involved in its regulation.

GSDMs could also serve as valuable biomarkers for cancer prognosis. Studies have shown that the expression pattern of GSDMB is closely associated with cancer progression, immune microenvironment, chemotherapeutic efficacy and prognosis. By analyzing 267 colorectal cancer samples, cytoplasmic GSDMB expression was found to be an independent indicator of good prognosis and correlated with 5-fluorouracil chemosensitivity. High GSDMB expression enhanced the sensitivity of cancer cells to 5-fluorouracil. Membrane and nuclear GSDMB-positive expression were associated with an increase in S100A8^+^ immune cells in the tumor invasive front, and nuclear GSDMB positivity was also associated with an increase in CD68^+^ macrophages in the TME. In addition, GSDMB^+^ immune cell density was associated with an increased proportion of neutrophils and a decreased proportion of lymphocytes and monocytes in the peripheral blood (65). The subcellular localization pattern of GSDMD is also closely associated with colorectal cancer progression and immune response. Immunohistochemical analysis of 178 colorectal cancer samples revealed that high expression of cytoplasmic GSDMD was an independently good indicator of prognosis and improved the efficacy of chemotherapy. Cytoplasmic GSDMD was associated with a reduced risk of distant metastasis, while nuclear GSDMD predicted deeper tumor infiltration. Membranous GSDMD was positively correlated with CD68^+^ macrophages in tumor center and CD8^+^ lymphocytes in tumor invasive front (66). The expression levels of specific GSDM family members in tumors may correlate with disease progression and response to therapy. Identifying these patterns could aid in stratifying patients for personalized treatment approaches, optimizing therapeutic efficacy (**Figure 3**).

### 5.1 Chemotherapy induced pyroptosis

Chemotherapy is confirmed to elicit pyroptosis in cancer cells. It was found that DOX reduced cell survival and induced a focal morphology in a dose-dependent manner. GSDME and caspase-3 were key regulators in this process. DOX treatment also resulted in increased ROS accumulation and JNK phosphorylation. It is shown that DOX-induced cellular pyroptosis in breast cancer cells occurs through a caspase-3-dependent pathway involving ROS/JNK signaling (67). Overexpression of lens protein with glutamine synthetase domain (LGSN) in gastric cancer stem cells has been found to be associated with poor patient survival and cancer progression. LGSN promotes therapeutic resistance by interacting with vimentin and inducing epithelial-to-mesenchymal transition (EMT). Genetic interference with LGSN inhibits tumor formation and restores sensitivity to chemotherapy not only by inhibiting gastric cancer stem cell stemness, but also by triggering GSDMD-mediated pyroptosis through activation of vimentin degradation/NLRP3 signaling (68). D2 dopamine receptor (DRD2) is a dominated member of D2-like receptors, improves drug sensitivity to paclitaxel (PTX) of breast cancer patients. Studies have shown that DRD2 modulates the TME by promoting M1 macrophage polarization and triggering GSDME-mediated pyroptosis. These findings highlight DRD2 as a potential prognostic biomarker and therapeutic target for breast cancer, offering insights into its role in regulating programmed cell death and immune modulation (69).

### 5.2 Radiotherapy induced pyroptosis

Irradiation-induced pyroptosis through activated caspase-3-mediated cleavage of GSDME is essential for radiosensitivity. GSDME was found to be downregulated in radioresistant nasopharyngeal carcinoma and associated with poor prognosis. Ovarian tumor family deubiquitinase 4 (OTUD4) enhanced radiosensitivity of nasopharyngeal carcinoma by promoting pyroptosis through deubiquitination and stabilization of GSDME (24). GSDME was found to be silenced in colorectal cancer (CRC) but expressed in surrounding normal tissues. GSDME/caspase-3 pathway mediates radiation-induced pyroptosis and sensitizes CRC cells to radiation. In animal models, GSDME expression reduces tumor volume and weight and enhances anti-tumor immunity by recruiting and activating NK cells. In addition, GSDME knockout mice were protected against radiation-induced tissue damage. These findings highlight the role of GSDME in radiosensitivity and link radiation to pyroptosis (70).

### 5.3 Immunotherapy induced pyroptosis

GSDMs, particularly GSDMD, facilitate the release of pro-inflammatory cytokines and DAMPs upon activation. This release recruits immune cells such as macrophages, DCs, and T-lymphocytes into the TME, enhancing the anti-tumor immune response. The increased immune cell infiltration can lead to improved tumor recognition and destruction, highlighting the potential of GSDMs in augmenting immunotherapeutic strategies. It was shown that cytotoxic T lymphocyte-associated antigen 4 (CTLA-4) blockade therapy improved the efficacy of head and neck squamous cell carcinoma (HNSCC) by inducing pyroptosis through GSDMs. CTLA-4 blockade activated CD8^+^ T cells and increased the levels of IFN-γ and TNF-α in the TME, which activated the STAT1/IRF1 axis to synergistically induce tumor cell pyroptosis and release large amounts of inflammatory substances and chemokines, thus improving the efficacy of CTLA-4 blockade therapy. This study reveals a new mechanism by which CTLA-4 blockade induces tumor cell pyroptosis by activating CD8^+^ T cells to release IFN-γ and TNF-α (71). Oncolytic parapoxvirus ovis (ORFV) was found to trigger the pyroptosis of GSDME-low tumor cells by decreasing ubiquitination of GSDME. Activation of GSDME elevated the proportion of CTLs in the tumor and enhanced the efficacy of ORFV therapy. In *in vivo* assays, ORFV preferentially accumulated in tumors after systemic delivery, triggering pyroptotic tumor killing and sensitizing immunologically “cold” tumors to checkpoint inhibition. This study highlights the critical role of GSDME-mediated pyroptosis in ORFV anti-tumor immunity and suggests a new strategy for combination cancer therapy (72). Splicing Factor 3b Subunit 1 (SF3B1) is overexpressed in ovarian cancer and is associated with low cytotoxicity immune cell infiltration. Inhibition of SF3B1 induced ovarian cancer cell pyroptosis and release of mitochondrial DNA, which activated macrophage polarization toward the M1 type. In a mouse model, the SF3B1 inhibitor pladienolide B enhanced the infiltration of cytotoxic immune cells and increased programmed death-ligand 1 (PD-L1) expression, potentiating the antitumor effect of αPD-L1. This study demonstrated that SF3B1 inhibition improved the immune microenvironment of ovarian cancer and synergized with immune checkpoint blockade (ICB) therapy, providing preclinical evidence for combination therapy. However, GSDMD expression in TME antigen-presenting cells (APCs) has been shown to correlate with immune checkpoint signatures and restrict anti-tumor immunity during PD-L1 inhibition. Mechanistically, GSDMD suppresses interferon-stimulated genes (ISGs) and impairs CD8+ T cell activation in a cGAS-dependent manner. Notably, genetic ablation of GSDMD in APCs enhances ISG expression and promotes CD8+ T cell-mediated anti-tumor responses. Furthermore, combined pharmacological inhibition of GSDMD-mediated pyroptosis and PD-L1 blockade has demonstrated synergistic effects in enhancing anti-tumor immunity. These findings underscore the therapeutic potential of targeting GSDMD in conjunction with immune checkpoint inhibitors to improve cancer immunotherapy outcomes (55).

### 5.4 Targeted therapy induced pyroptosis

It was established that BRAF and MEK inhibitors (BRAFi + MEKi) enhance anti-tumor immune responses by promoting GSDME cleavage and HMGB1 release, triggering pyroptosis in BRAF V600E/K mutant melanomas. GSDME-deficient melanomas exhibit blocked release of HMGB1, reduced tumor-associated T-cells and activated DC infiltration, and more frequent tumor regrowth after drug withdrawal. Drug-resistant melanomas lack pyroptosis markers but are sensitive to pyroptosis-inducing chemotherapy. This study confirms the role of BRAFi + MEKi induced pyroptosis in anti-tumor immunity and suggests new therapeutic strategies against drug-resistant melanoma (73). IBI315, a recombinant fully human IgG1 bispecific antibody targeting both programmed death-1 (PD-1) and Her2, triggers GSDMB-mediated pyroptosis in tumor cells, leading to the activation and recruitments of T cells. The activated T cells secret IFN-γ enhancing GSDMB expression and establishing a positive feedback loop of T cell activation and tumor cell killing (74). The multi-CDK inhibitor AT7519 was able to block the cell cycle in G1-S and G2-M phases by inhibiting CDK1/2 phosphorylation and induced endogenous apoptosis and pyroptosis of glioblastoma multiforme (GBM) via caspase-3-mediated GSDME cleavage (75). Sorafenib, a multikinase inhibitor for hepatocellular carcinoma (HCC), induces pyroptosis in macrophages via caspase-1 activation, triggering the release of IL-1β and IL-18, which are critical for NK cell activation and subsequent tumor cell killing. Additionally, sorafenib downregulates MHC-I expression on tumor cells, potentially reducing their responsiveness to ICIs while enhancing NK cell-mediated cytotoxicity (76).

### 5.5 Nano-based therapy induced pyroptosis

Researchers have devised a new way to enhance cancer immunotherapy by modulating tumor glycometabolism. GOx-Mn nanoparticles were developed using a biomineralization-like approach containing hybridizing nanozymes and glucose oxidase (GOx). GOx functions as a crucial metabolic enzyme that catalyzes glucose to gluconic acid and H_2_O_2_ in tumor sites, though its oxygen-dependent nature limits its efficiency in hypoxic tumor environments. This limitation is overcome by coupling GOx with Mn-containing nanozymes, which catalyzes H_2_O_2_ to generate O_2_, thereby maintaining continuous glucose metabolism at tumor site. The consumption of glucose from tumor cell by GOx-Mn nanoparticles leads to pyroptosis and more PD-L1 expression. This approach, combined with anti-PD-L1 therapy, significantly inhibited tumor growth and prolonged mouse survival. In addition, it produces an immune memory effect that prevents tumor recurrence and metastasis (77). Nanocarriers containing the natural compound Aloe-emodin (AE) were able to release AEs in the acidic microenvironment of tumors, which significantly increased intracerebral distribution and tumor tissue accumulation via transferrin (Tf) and polyethylene glycol-poly (lactic-co-glycolic acid) (PEG-PLGA) modifications, and enhanced the pyroptosis effect of glioblastoma (GBM) while activating anti-tumor immunity and reducing AE-related toxicity (78). A study has developed a smart ROS/glutathione (GSH) dual-responsive nano-prodrug containing the drug paclitaxel (PTX) and the photosensitizer purpurin 18. This nano-prodrug achieves the optimal release of the loaded drug through the dual response to ROS and GSH in the TME, while the loaded drug paclitaxel (PTX) and the photosensitizer purpurin 18 (P18), after being irradiated by laser light, triggered GSDME-induced pyroptosis in tumor cells through chemo-photodynamic effects. At the same time, these pyroptotic tumor cells release DAMPs to activate adaptive immunity, enhance ICB efficiency, promote tumor regression and generate immune memory to prevent recurrence. This mechanism suggests a potential strategy to trigger pyroptosis and enhance ICB efficiency by synergistically inducing GSDME-induced pyroptosis (79).

### 5.6 Photodynamic therapy induced pyroptosis

Researchers explored a mitochondria-targeted photodynamic therapy (PDT) to enhance anti-tumor immunity in microsatellite-stable colorectal cancer (MSS-CRC). The photosensitizer IR700DX-6T (a photosensitizer targeting the mitochondrial translocation protein) was utilized to induce pyroptosis, which generates ROS and activates caspase-3-mediated GSDME cleavage. This process sensitizes MSS-CRC cells to PD-1 blockers. In addition, disruption of GSDME methylation using decitabine improved the efficacy of PDT and further enhanced the antitumor response when combined with PD-1 inhibitors. This study suggests that mitochondria-targeted PDT may improve the efficacy of immunotherapy against CRC (80).

### 5.7 Others

Metformin, a classic type 2 diabetes treatment drug, was found to activate Bax and cytochrome c release by enhancing the AMPK/SIRT1/NF-κB signaling pathway, triggering caspase-3-mediated GSDME cleavage, which leads to pyroptosis. In addition, metformin induced mitochondrial dysfunction, which further promoted the activation of the AMPK/SIRT1 pathway and drove pyroptosis, revealing a novel mechanism of metformin in antitumor effect by inducing pyroptosis (81). The potential clinical applications of GSDM pathway activators or inhibitors, particularly in combination with immunotherapy or chemotherapy, hold significant promise in improving cancer treatment outcomes. Activators of GSDM pathways, such as small molecules, epigenetic modulators, or nanoparticles, can induce pyroptosis, leading to the release of tumor antigens and DAMPs, which enhance DC maturation and antigen presentation. This process can amplify the efficacy of ICIs by converting immunosuppressive tumors into immunologically active tumors. On the other hand, inhibitors of GSDM pathways may be therapeutically relevant in cancers where chronic inflammation driven by excessive pyroptosis promotes tumor progression. For instance, blocking GSDMD activation in inflammation-associated cancers, such as colorectal or pancreatic cancer, could reduce the recruitment of immunosuppressive cells, thereby mitigating pro-tumorigenic effects. Moreover, pyroptosis induction can lead to adverse effects through off-target inflammation or damage to healthy tissues. The release of pro-inflammatory cytokines during pyroptosis can exacerbate systemic inflammation, leading to cytokine release syndrome (CRS) or autoimmune-like responses (82). Additionally, pyroptosis-induced tissue damage in non-tumor sites has been observed, raising concerns about its safety in clinical applications (83). To mitigate these risks, several strategies have been proposed. First, tumor-specific delivery of pyroptosis inducers using targeted nanoparticles or tumor-selective promoters (e.g., survivin or hypoxia-inducible promoters) can minimize off-target effects. Second, the co-administration of anti-inflammatory agents, such as IL-6 or IL-1β inhibitors, can dampen unwanted systemic inflammation without compromising the anti-tumor effects of pyroptosis. Third, optimizing the dosage and timing of pyroptosis inducers in combination with existing therapies, such as ICIs or chemotherapy, can achieve a therapeutic balance by enhancing anti-tumor immunity while minimizing collateral damage. Finally, biomarker-driven patient stratification, based on GSDM expression levels, inflammasome activity, and tumor immune composition, can identify patients most likely to benefit from pyroptosis-targeted therapies, reducing the risk of adverse effects.

## 6 Conclusion and perspectives

This review provides an overview of the role of GSDMs and their mechanisms in tumorigenesis and development and anti-cancer therapy, especially the role of GSDMs-induced pyroptosis in this context. Although, the role of GSDMs in pyroptosis is well established, GSDMs can also act independently of this process. It was found that aggregation of GSDM-NT does not necessarily predict pyroptosis, and that cells may survive after GSDM-NT formation of pores due to plasma membrane (PM) repair mechanisms, weak inflammasome activation, and oxidized lipid stimulation (84). In addition, available evidence suggests that GSDMs can even induce pyroptosis without cleavage (28). GSDMs not only affect the tumor microenvironment, but also interact with other cell death pathways, exhibiting a complex regulatory network. Of course, there are still many unknowns about GSDMs that need to be revealed urgently, including whether oligomeric pores are formed immediately after GSDMs-NT insertion into the membrane or are first assembled in the cytoplasm and then transported into the lipid bilayer as a single unit? Does the protein modification of GSDMs affect their membrane insertion or organelle specificity? How do cellular contents released through the pore differ between immune and cancer cells? And what are the implications for anti-tumor immunity? More studies are expected in the future to explore the mechanism of regulation of GSDMs activity and interactions with other cells and cell death pathways.
